# Processing of Lipid Nanodispersions into Solid Powders by Spray Drying

**DOI:** 10.3390/pharmaceutics14112464

**Published:** 2022-11-15

**Authors:** Denise Steiner, Leonie V. Schumann, Heike Bunjes

**Affiliations:** 1 Institut für Pharmazeutische Technologie und Biopharmazie, Technische Universität Braunschweig, Mendelssohnstraße 1, 38106 Braunschweig, Germany; 2 Zentrum für Pharmaverfahrenstechnik (PVZ), Technische Universität Braunschweig, Franz-Liszt-Straße 35a, 38106 Braunschweig, Germany

**Keywords:** formulation technology, solid lipid nanoparticles, lipid nanoemulsions, poorly water-soluble drug, nanotechnology

## Abstract

Spray drying is a promising technology for drying lipid nanodispersions. These formulations can serve as carrier systems for poorly water-soluble active pharmaceutical ingredients (APIs) that are loaded into the lipid matrix to improve their bioavailability. Once the API-loaded nanocarriers have been further processed into solid dosage forms, they could be administered orally, which is usually preferred by patients. Various solid lipids as well as oils were used in this study to prepare lipid nanodispersions, and it was shown that their nanoparticulate properties could be maintained when lactose in combination with SDS was used as matrix material in the spray-drying process. In addition, for lipid nanoemulsions loaded with fenofibrate, a good redispersibility with particle sizes below 300 nm at a lipid content of 26.8 wt.% in the powders was observed. More detailed investigations on the influence of the drying temperature yielded good results when the inlet temperature of the drying air was set at 110 °C or above, enabling the lactose to form an amorphous matrix around the embedded lipid particles. A tristearin suspension was developed as a probe to measure the temperature exposure of the lipid particles during the drying process. The results with this approach indicate that the actual temperature the particles were exposed to during the drying process could be higher than the outlet temperature.

## 1. Introduction

The increasing number of newly developed active pharmaceutical ingredients (APIs) that are poorly water-soluble is a well-known challenge that formulation specialists have been facing in recent years [1]. The high lipophilicity and/or highly stable crystal lattice of these substances often lead to a very low bioavailability [2]. To overcome this drawback, various formulation strategies have been developed. A very promising approach is the loading of these APIs into lipid nanocarriers such as nanosuspensions or -emulsions [3]. These lipid carriers are biocompatible and, therefore, suitable for different routes of administration [4,5]. After preparation, such lipid carrier systems are in the liquid state and can, therefore, be used easily for parenteral and dermal drug delivery, as already done in many applications. For oral use, a solid dosage form is more suitable and enjoys much higher patient acceptance [6]. Studies on the oral bioavailability of poorly water-soluble APIs in lipid nanodispersions revealed a positive effect on the absorption of the APIs due to the presence of the lipid [7,8] and, in addition, a reduction in the so-called food effect [9]. To formulate a solid drug delivery system for the API-containing lipid particles, a further processing of the API-loaded nanodispersions into dry powders is required. These powders could then be administered to the patient directly or serve as an intermediate product for the formulation of granules or tablets [10,11].

One possibility to further process lipid nanodispersions into lipid-containing powders is by spray drying. This drying process is well established and used in various disciplines. The spray-drying behavior of lipid nanoemulsions has already been studied in some detail, with formulations dried with and without the addition of a matrix material. Christensen et al., for example, prepared fractionated coconut oil emulsions (size x_50_ = 820 nm) with different HPMC (hydroxypropyl methyl cellulose) types and spray-dried these formulations without adding additional matrix material. According to their results, this allowed an embedding of up to 40% lipid in the dry powder mass, with only a slight increase in droplet sizes after redispersion of the powders [12]. In another study of these authors, various matrix materials were added to the emulsion before spray drying to increase the powder density before tableting of lipid-containing but API-free powders. This resulted in solid dosage forms with a lipid content of up to 20% [11]. APIs such as intraconazole [13] or 5-PDTT (5-phenyl-1,2-dithiole-3-thione) [14] have also been incorporated into lipid emulsions and embedded in matrices by spray drying. In vitro and in vivo studies conducted with the resulting API-containing powders showed improved bioavailability of both APIs compared to the unformulated API or a previous dosage form prepared with cyclodextrins.

While the physical state of emulsion droplets is not affected by high temperatures during the spray dying process, drying of solid lipid nanoparticles could be more challenging because they may melt during the process. Foundational studies by Masters on the spray drying of particle-containing formulations indicated that the temperature exposure of the product particles during drying is approx. 15 to 25 °C below the outlet temperature of the drying air (measured between the drying chamber and the process unit for particle separation) [15]. In the course of investigations on the spray-drying behavior of protein solutions, this perception was modified and it is currently assumed that the outlet temperature describes the maximum temperature the formulations are exposed to upon drying [16,17]. The first spray-drying studies with drug-free dispersions of solid lipid particles from different lipid types, such as tristearin and Compritol, indicated that such particles could be embedded in matrices without losing their nanoparticulate properties. The authors of these studies assumed that the temperatures to which the particles were exposed to were well below their melting temperatures and, thus, did not have an effect on the physical state of the lipids during drying [18,19]. In studies dealing with the spray drying of Compritol particles loaded with rapamycin, agglomeration of the particles after redispersion of the powders was observed. This was considered to be a consequence of suboptimal process conditions, although a good reproducibility was shown [20].

The current study started with investigations on tristearin nanodispersions initially focusing on the identification of a suitable matrix material for embedding the lipid particles upon spray drying, as well as an optimized formulation for preparing the nanodispersions by high-pressure homogenization. To evaluate the transferability of the optimized formulation to other lipid nanocarrier systems, various dispersions of oils and solid lipids were spray-dried and the redispersibility of the lipid-containing powders in water was tested. Nanoemulsions loaded with the model API fenofibrate were also included in these investigations. In the last part of this study, the time- and formulation-dependent polymorphic transformation of tristearin nanoparticles was used to further investigate the effect of the drying temperature on the physical state of the particles [21,22]. While triglycerides are known to crystallize first in the metastable α-polymorphic form and then transfer to the more ordered and stable β-modification [23,24], a suspension containing mainly nanoparticles in the β-polymorphic form was spray-dried and the content of particles that had melted during the process was evaluated in dependence on the drying temperature.

## 2. Materials and Methods

### 2.1. Materials

Various solid lipids as well as oils were used for the preparation of lipid nanodispersions. Solid glycerides: Compritol (mono-, di-, and triesters of behenic acid, Compritol 888; Gattefossé, Saint-Priest Cedex, France), tristearin (Dynasan 118), tripalmitin (Dynasan 116), and trimyristin (Dynasan 114; all from Hüls/Condea, Witten, Germany; all kind gifts from the manufacturer). Oils: Miglyol (medium-chain triglycerides, Miglyol^®^812; Caesar&Loretz, Hilden, Germany), refined soybean oil (Roth, Karlsruhe, Germany), and refined rapeseed oil (Caelo, Hilden, Germany). Lipid dispersions were stabilized with the polymers PVA (polyvinyl alcohol, Mowiol 3-83; Kuraray Europe, Hattersheim, Germany) or HPMC (hydroxypropyl methyl cellulose, Pharmacoat 606; Shin-Etsu Chemical Eo., Tokyo, Japan; kind gift from Harke Pharma, Mülheim an der Ruhr, Germany) and the surfactant SDS (sodium dodecyl sulphate; Roth, Karlsruhe, Germany). The following matrix materials were used during spray drying: lactose (lactose monohydrate; Meggle, Wasserburg am Inn, Germany; kind gift from the manufacturer), mannitol (D-mannitol; Sigma-Aldrich, Taufkirchen, Germany), and sucrose (D(+)-saccharose; Roth, Karlsruhe, Germany). The poorly water-soluble API fenofibrate (FENO; Novartis Pharma AG, Basel, Switzerland; kind gift) was used as a model substance. Tetrahydrofuran (THF; Sigma-Aldrich, Taufkirchen, Germany) was used for characterization purposes. Bidistilled water was used in all experiments.

### 2.2. Preparation of Lipid Nanodispersions

Lipid nanodispersions were prepared by high-pressure homogenization (Microfluidizer M110S; Microfluidics, Westwood, MA, USA) at room temperature (oils) or a process temperature of approx. 10 °C above the melting temperature of the respective solid lipid. All lipid nanodispersions contained 10 wt.% lipid, 5 wt.% polymer (PVA or HPMC), and 0.25 wt.% SDS. A pre-emulsion was first prepared from the liquid (melted) lipid and the aqueous phase, containing the stabilization polymer and SDS, with an Ultra-Turrax (IKA-Werke, Staufen im Breisgau, Germany) for 2 min at 13,000 rpm. Subsequently, the formulations were high-pressure-homogenized at 800 bar for 10 cycles. Formulations containing Compritol, tristearin, and tripalmitin (lipid nanosuspensions) were cooled to allow droplet crystallization (ice bath, 30 min). The oil-containing formulations were cooled to room temperature (lipid nanoemulsions). Trimyristin nanodispersions are known to form supercooled droplets, which allow the trimyristin nanoparticles to exist in liquid and solid states at room temperature. To obtain a trimyristin nanosuspension, the respective emulsion was cooled in an ice bath to allow crystallization of the trimyristin droplets. The particles remained solid when subsequently heated to room temperature. Trimyristin droplets were retained in the liquid state after high-pressure melt homogenization by cooling to room temperature after preparation [25]. Until spray drying, all lipid formulations were stored in sealed glass vessels at 20 °C.

### 2.3. Loading of Lipid Nanodispersions with API

Fenofibrate (FENO) was loaded into the lipid nanodispersions using the passive loading method introduced by Rosenblatt and Bunjes [26], aiming for a maximum API load in the lipids. In this method, poorly water-soluble APIs are mixed with the lipid carriers. During the incubation period, the API molecules are then embedded into the lipids, while overload is prevented due to the equilibrium with the crystalline particles.

The lipid nanodispersions (15 mL) were incubated with FENO microparticles (approx. 0.3 g, starting material) in glass vials for 5 days at 20 °C under stirring. Afterward, the formulations were filtered through a 0.45 µm PVDF filter (Roth, Karlsruhe, Germany) to remove the excess API crystals from the dispersions. Prior to spray drying, all formulations were stored overnight at 20 °C.

### 2.4. Spray Drying of Lipid Nanodispersions

Spray drying of the lipid nanodispersions was carried out using a Büchi B-191 (Büchi AG, Flawil, Switzerland) in a co-current air flow mode. A peristaltic pump transported the lipid-matrix former formulation to the two-fluid nozzle that had an orifice diameter of 0.7 mm, with a volume flow of 2 mL min^−1^. The atomization pressure was set to 2.0 bar and the air flow rate was between 350 and 360 m^3^ h^−1^. Particles were separated from the air flow using a high-performance cyclone and collected in a glass vessel below this. The inlet temperature T_in_ was set between 70 and 200 °C and the resulting outlet temperatures T_out_ were measured between the spray tower and the particle separation unit. Based on the experimental setup, it can be assumed that the temperature of the cyclone and the collection vessel was as high as T_out_ or lower.

Prior to spray drying, the nanosuspensions and -emulsions were mixed with the matrix former solution containing 5 wt.% mannitol, sucrose, or lactose. The surfactant SDS was added to this solution at concentrations between 0.0 and 1.0 wt.%. The lipid content in the dried powders ranged from 10.9 wt.% to 42.6 wt.%. Before the drying process of the liquid formulation containing the lipid and the matrix material started, the spray dryer was heated to the required temperature and distilled water was sprayed for at least 5 min.

### 2.5. Characterization of Lipid Dispersions and Powders

#### 2.5.1. Particle Size Analysis

All particle sizes were measured by laser diffraction using a LA-960 (Horiba Scientific, Kyoto, Japan). Lipid nanosuspensions and -emulsions were measured on the day of their preparation when they had reached room temperature. Lipid-containing powders (0.2 g) were dissolved in 0.4 mL of water for one hour prior to measurement. To achieve an appropriate particle concentration for the size measurement, all samples were diluted in distilled water. Each sample was measured in triplicate and the Mie-theory-based evaluation model was used to calculate the volume distribution. A refractive index of 1.46 (absorption index of 0.01) was assumed for the lipid particles and 1.33 for water.

#### 2.5.2. Viscosity Measurement

Dynamic viscosities of lipid nanodispersions were measured using the HAAKETM RheoStress 6000 rotational viscometer (Thermo Fischer Scientific, Waltham, MA, USA). A DG41 double-gap Searle measurement system was used for the measurements, which were performed with 10 mL of sample at shear rates between 5 and 1000 s^−1^. The gap height was set to 5.1 mm and all samples were measured at 25 °C. All formulations showed Newtonian flow properties at the shear rates under investigation; thus, the dynamic viscosity was given independent of the shear rates applied.

#### 2.5.3. X-ray Diffraction

The crystallinity of the spray-dried powders was characterized by XRD (X-ray diffraction). The powders were placed in a sample holder immediately after spray drying and measured using a PW3050/60 MPD Goniometer equipped with a Pre FIX X’Celerator detector (PANalytical, EA Almelo, The Netherlands). All samples were measured at room temperature with a step size of 0.05° 2θ in a range of 5° to 45° 2θ.

#### 2.5.4. Differential Scanning Calorimetry

The polymorphic state of the lipid nanoparticles was analyzed by differential scanning calorimetry (DSC) using a DSC3+ equipped with a FRS6+ sensor (Mettler Toledo, Columbus, OH, USA). All samples were weighed into 40 µL aluminum crucibles, which were cold-welded. When measuring lipid nanosuspensions, an 18 µL sample was pipetted into the crucibles. Approx. 5 mg of the sample was used when measuring dried powders. The lipid nanosuspensions were measured after a storage time of 1 day or 6 months and the dried powders were analyzed immediately after drying. Measurements to determine the melting events were performed at a heating rate of 10 °C min^−1^ from 20 °C to 85 °C. The crystallization properties of the trimyristin droplets were determined at a cooling rate of −10 °C min^−1^ from 25 °C to 0 °C.

#### 2.5.5. Residual Moisture

The residual moisture of the powders was determined on selected samples. Immediately after spray drying, the powder was weighed into a glass vial and dried in an oven at 95 °C for 24 h. After the samples had cooled to room temperature, the dried powder was weighed again and the residual moisture calculated.

#### 2.5.6. Determination of API Content

The FENO content in the lipid dispersions was determined by UV-Vis spectroscopy (SPECORD 210 PLUS; Analytic Jena, Jena, Germany). The loaded lipid nanoemulsions and -suspensions were dissolved in a THF/water mixture (ratio 9:1, *v*/*v*) and diluted with this solvent mixture until a linear correlation of the absorption with the FENO concentration could be confirmed. A calibration curve was prepared prior to measurement and the pure solvent was analyzed as the reference sample. The absorbance of the dissolved, unloaded lipid dispersions was measured and subtracted from the value of the FENO-containing sample. The measurements were performed at a wavelength of 287 nm.

#### 2.5.7. Scanning Electron Microscopy

Images of selected lipid-containing powders were taken with a scanning electron microscope (SEM). Samples were prepared by sputtering the powders with gold at 5 mA for 4 min in a Balzers Union SCD 030 sputter device. Images were then taken using a Helios C4 CX (FEI Deutschland GmbH, Frankfurt am Main, Germany) under high vacuum at a voltage of 2.00 kV.

## 3. Results and Discussion

### 3.1. Impact of Matrix Material on the Redispersibility of a Tristearin Nanosuspension

Initial drying experiments were carried out with a tristearin nanosuspension. During high-pressure homogenization, the suspension was stabilized with the polymer PVA in combination with the surfactant SDS. The mean particle size of the tristearin suspension before drying was x_50_ = 117 nm. All formulations described in this section were dried at T_in_ = 110 °C and with a lipid content of 21.5 wt.% in the dried powders.

#### 3.1.1. Use of Different Matrix Materials

To prevent agglomeration or aggregation of the lipid nanoparticles during drying, various matrix materials were added to the tristearin nanosuspension prior to spray dying. Three of the most commonly used matrix materials, mannitol, sucrose, and lactose, were applied to evaluate their ability to maintain the nanoparticulate properties of the tristearin nanosuspension in the dried powder. When the surfactant SDS was added to the matrix solution, it was carried out at a concentration of 0.5 wt.% referred on the total weight of the matrix solution.

**Figure 1:**
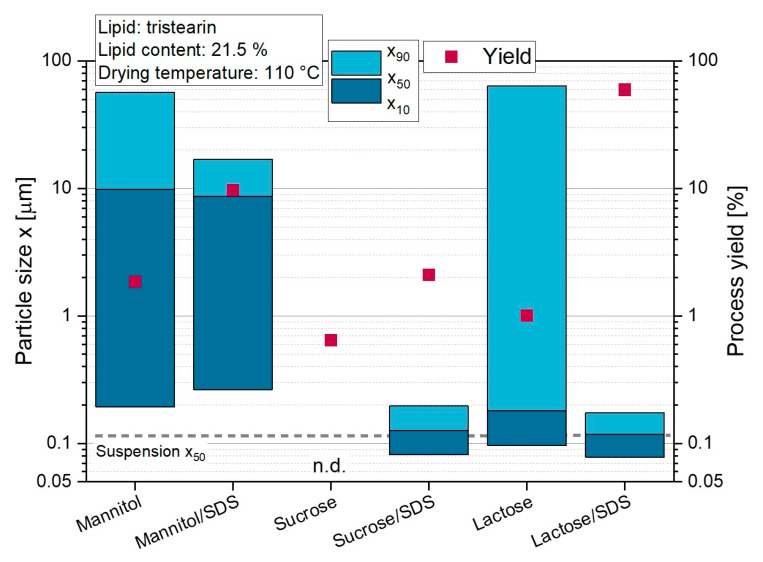
Effect of the matrix material on the size of tristearin nanoparticles after redispersion of lipid-containing powders in water and on the yield of the spray-drying process (n.d. = not determinable).

The sizes of the lipid particles redispersed from the powders containing only mannitol and lactose as matrix material indicated a high agglomeration tendency (Figure 1). Redispersion of the sucrose-embedded tristearin particles was even impossible. The wettability of the lipid-containing powders could be improved by adding the surfactant SDS to the matrix solution. Thus, particles with a median particle size of 118 nm and 126 nm could be obtained when lactose/SDS and sucrose/SDS were used, respectively, but no improved redispersibility was shown when SDS was added to mannitol. These results indicated that there might be a correlation between the physical state of the matrix materials after spray drying and the redispersibility of lipid nanoparticles from the powders. Previous studies showed that lactose and sucrose formed an amorphous powder, while mannitol crystallized during spray drying [10,27]. Thus, it was assumed that the poor redispersibility of the mannitol-containing powders, despite the addition of SDS, was caused by the crystalline state of the matrix material. This assumption was supported by results of an investigation performed later in this study that focused on the variation in the drying temperature (Section 3.5, with matrix material: lactose/SDS).

In order to evaluate the spray dying process, the process yield was investigated in more detail for these experiments. The highest yield of 60 % was achieved for the formulation with the matrix material lactose/SDS. The lowest process yields were obtained when sucrose was used as matrix material, which was attributed to the strong deposition of powder on the glass walls of the cyclone. It is assumed that this was caused by the distinctly lower glass transition temperature of amorphous sucrose (T_g,sucrose_ = 62 °C), compared to amorphous lactose (T_g,lactose_ = 101 °C) [28]. While T_out_ was approx. 64 °C in these experiments (T_in_ = 110 °C) and it is likely that the temperature of the cyclone walls was approx. as high as T_out_ [29], this resulted in adhesion of the sticky sucrose formulation on the glass. Based on these results, lactose/SDS was chosen as matrix material in the following experiments.

#### 3.1.2. Variation in Surfactant Content in Lactose Matrix

**Figure 2:**
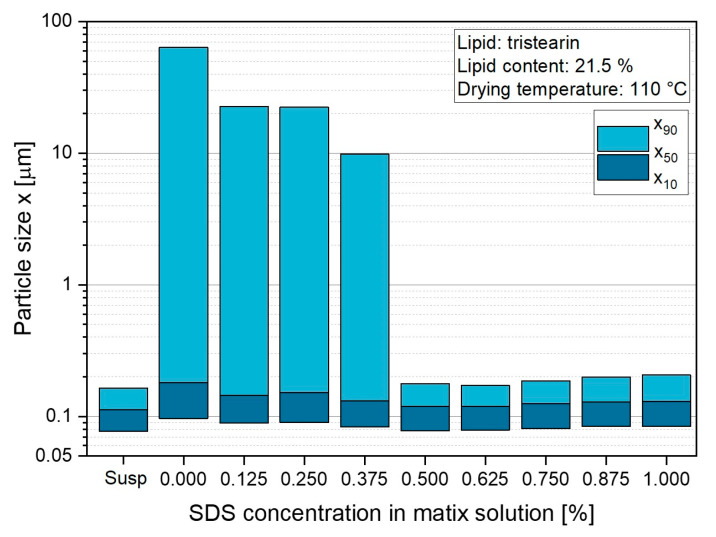
Influence of the SDS concentration in the lactose matrix solution as reflected by the size of the tristearin particles redispersed from the dried powders compared to that of the suspension (susp) before drying.

The experiments described above already demonstrated that the presence of the surfactant SDS in the lactose matrix had a significant positive effect on the redispersibility of the tristearin-containing powders. To further evaluate the SDS content required in the formulation to maintain the size of the tristearin particles after drying, SDS was added to the lactose solution (5 wt.%) at different concentrations (Figure 2). As already shown above, large agglomerates with x_90_ = 63 µm were obtained when no SDS was added to the lactose solution. The redispersibility of the powders improved slightly by adding up to 0.375 wt.% SDS to the solution, but particles with x_90_ ≥ 10 µm were still obtained for these samples. A good redispersibility of the tristearin-containing powders was only observed when 0.5 wt.% or more SDS was added to the matrix solution. However, the results also indicated that an increase in the SDS concentration above 0.5 wt.% did not result in a narrower size distribution. Therefore, the lipid formulations described in the following were all mixed with a matrix former solution containing 5 wt.% lactose and 0.5 wt.% SDS prior to spray drying.

### 3.2. Influence of Particle Stabilization on Drying Properties of Tristearin Nanosuspensions

In addition to the matrix former solution added to the lipid nanosuspension directly before spray drying, the particle stabilization additives selected for the preparation of the lipid nanosuspension during high-pressure homogenization might also affect the quality of the final powder. Thus far in this study, the polymer PVA was used together with the surfactant SDS to stabilize the tristearin particles during preparation. However, in a previously conducted stabilizer screening in a dual centrifuge, further additives suitable for the stabilization of tristearin nanoparticles were identified [30]. One of the most promising formulations was the combination of the polymer HPMC with the surfactant SDS. In the following, the spray-drying properties of the nanosuspension formulated with HPMC/SDS during high-pressure homogenization were compared to the lipid-containing powders prepared from the tristearin suspension stabilized with PVA/SDS. In addition, the maximum possible lipid content in the powders was determined, which still allowed a total redispersion of the lipid particles in water.

After preparation of the lipid nanosuspensions, approx. 20 nm larger particles (x_50_) were obtained for the HPMC/SDS-stabilized suspension (x_50_ = 117 nm) than for the formulation stabilized with PVA/SDS. This is believed to be due to the different weight-average molecular weights of the polymers (M_W,PVA_ = 14.0 kg mol^−1^ and M_W,HPMC_ = 35.6 kg mol^−1^, according to the manufacturers), resulting in higher viscosities of the suspension prepared with HPMC/SDS (Table 1). It can be assumed that the polymer molecules were not only located at the particle interfaces, but also in the aqueous phase of the suspensions, causing the different viscosities. After mixing the nanosuspensions with the matrix former solution (lactose/SDS) before spray-drying (lipid content in powders: 21.5 wt.% and 31.5 wt.%), the differences in the viscosities decreased clearly. Therefore, it was assumed that the only slightly different viscosities of the diluted formulations could be neglected and did not have a major influence on the droplet formation during the spray-drying process.

**Figure 3:**
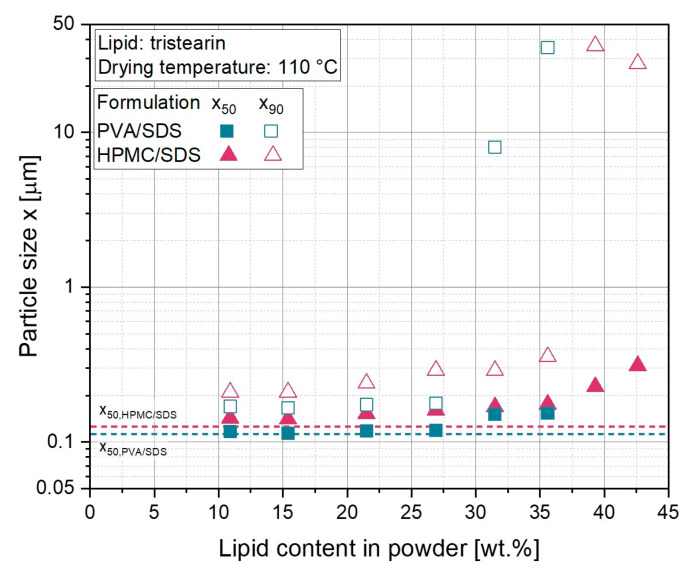
Particle sizes after redispersion of powders with different contents of tristearin nanoparticles and with two different particle stabilization systems during high-pressure homogenization: PVA/SDS and HPMC/SDS. The particle size of the original suspensions is indicated by the dashed lines.

The sizes of the differently (HPMC/SDS or PVA/SDS) stabilized tristearin particles redispersed from the powders showed no major differences in terms of particle size as compared to those prior to spray drying, up to a lipid content in the powders of 26.8 wt.% (Figure 3). However, once the lipid content was further increased, the redispersibility of the powders containing lipid particles stabilized with PVA/SDS deteriorated, indicated by x_90_ > 8 µm. In contrast, good redispersibility was obtained for particles stabilized with HPMC/SDS up to a lipid content of 35.6 wt.%. Similar correlations were observed when tristearin particles were embedded in a polymer matrix for the preparation of orodispersible films (ODFs) [30]. On the one hand, this showed that the composition of the dispersions prepared by high-pressure homogenization did have an influence on the subsequent processes. On the other hand, it was found that the presence of HPMC in the formulation resulted in better embedding of tristearin particles in the solid matrix, and, thus, higher lipid contents could be realized in the dried powders before agglomeration occurred. However, the process yield was also influenced by the additives used to stabilize the particles during high-pressure homogenization. The yield reduced from approx. 60 % for the suspension formulated with PVA/SDS to 10 % when the particles were formulated with HPMC/SDS (lipid content for both formulations 26.8 wt.%, data not shown). Whether the slightly higher viscosities during spray drying of the HPMC-containing formulations actually had such a strong influence on the process yield could not be conclusively clarified. In order to be able to make a definite statement, further studies would have to be carried out with various polymers.

### 3.3. Embedding of Different Lipid Nanosuspensions and -Emulsions in Lactose/SDS Matrix

The previous experiments showed that a tristearin suspension could be successfully embedded in a lactose/SDS matrix by spray drying, with no agglomeration occurring for lipid contents up to 26.8 wt.% (stabilization during high-pressure homogenization: PVA/SDS). The extent to which these results could be transferred to dispersions of other solid lipids or oils was investigated in the following. In addition to tristearin, particles of three other solid lipids, Compritol, tripalmitin, and trimyristin, were embedded in the lactose matrix. The transferability of the formulation to lipid nanoemulsions was investigated using rapeseed oil, soybean oil, Miglyol, and trimyristin. As mentioned above, trimyristin nanoparticles can form a supercooled melt and, thus, exist in both solid and liquid states at room temperature [25]. Therefore, trimyristin was spray-dried in the form of a nanosuspension and a nanoemulsion.

**Figure 4:**
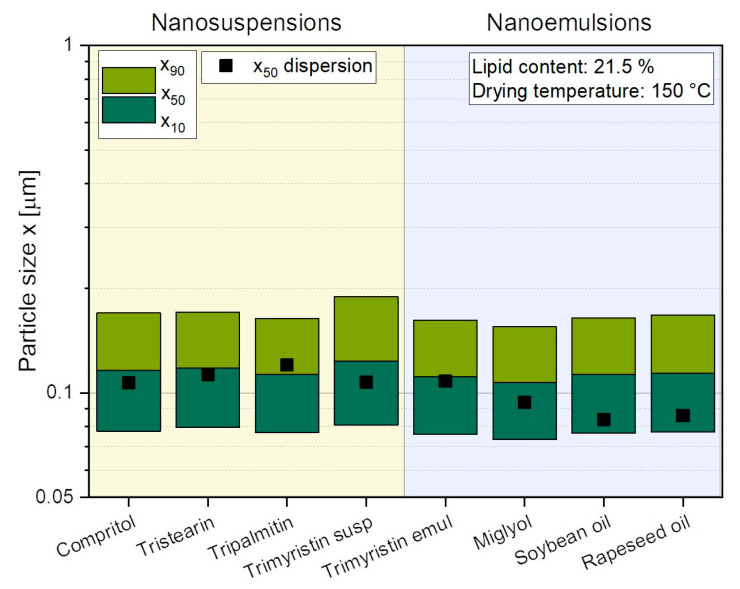
Particle size distributions after redispersion of spray-dried powders containing various lipid nanosuspensions and -emulsions. Trimyristin was embedded in the powders as suspension (susp) and emulsion (emul). The x_50_ values of the original dispersions before spray drying are also given for comparison.

Using the additives PVA/SDS to stabilize the nanodispersions during high-pressure homogenization, nanosuspensions as well as -emulsions with particle sizes between x_50_ = 85 nm and 120 nm were obtained (Figure 4). These lipid dispersions were then spray-dried and the powders redispersed in water before size measurement. The resulting particle sizes showed that all lipid-containing powders (lipid content in powder 21.5 wt.%) could be successfully redispersed in water, with only an increase in particle size x_50_ of at maximum 29 nm (for soybean oil) and no x_90_ value above 200 nm.

### 3.4. Investigation of Drug-Loaded Lipid Nanodispersions

**Figure 5:**
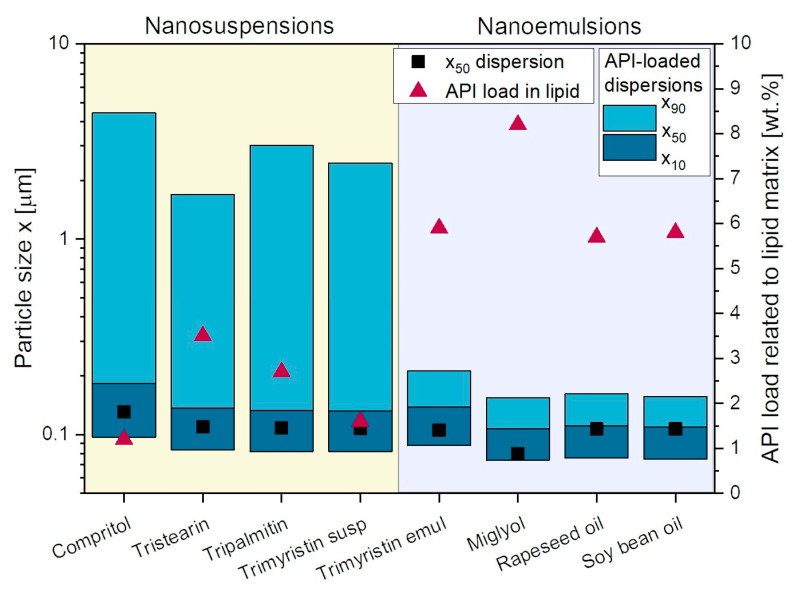
Particle sizes of lipid nanodispersions loaded with FENO and loadability of FENO in lipid nanodispersions related to the lipid matrix. The x_50_ values of the unloaded lipid nanodispersions are also displayed.

Lipid nanocarriers are used in pharmaceutical formulation development to improve the bioavailability of poorly water-soluble APIs. To evaluate whether spray drying of API-containing lipid carriers could be successful, the lipid nanodispersions (stabilized with PVA/SDS) were first loaded with FENO and the particle sizes of the API-loaded dispersions as well as the loadability of FENO into the lipid nanodispersions were determined (Figure 5).

Looking first at the lipid nanoemulsions, the results indicated that upon loading the lipid droplets with FENO, the nanoparticulate properties of the initial emulsions were maintained. A negligible increase in particle size was measured when rapeseed oil and soybean oil were loaded with FENO. The increase in particle size after API-loading was somewhat more pronounced for Miglyol and the tristearin emulsion, but did not exceed 30 nm. As for the drug loading related to the lipid matrix, the highest loading capacities of 8.2 wt.% FENO in the lipid matrix were obtained for the Miglyol emulsion, while they were very similar for the other three oils under investigation ranging from 5.7 to 5.9 wt.%.

Significantly different results were obtained when lipid suspensions were loaded with FENO. Particle agglomeration was evident after loading the lipid nanoparticles with FENO, indicated mainly by the high x_90_ of all suspensions. It is assumed that the explanation of these completely different behaviors of the API-containing lipid nanoemulsions and -suspensions is based on the different physical states of the lipids. During incubation, the API molecules could be loaded into the liquid matrix of the droplets, whereas when the crystalline lipid particles were loaded with the API, it was mainly located at the particles interface, where the stabilizing polymer resides [31,32]. Therefore, the agglomeration of the particles is assumed to be caused by some kind of competitive reaction of the polymer PVA and the API at the particle surface, which may have caused displacement of the polymer, but the exact mechanism leading to this particle agglomeration or aggregation has to be further investigated in future studies. This phenomenon can be avoided if particle stabilization is further optimized, as successful API loading of lipid nanoparticles has already been demonstrated for several APIs [3]. The difference in the physical state of the lipid also resulted in a significantly lower API load of the lipid suspensions, where the highest loading capacity of 2.7 wt.% was detected for tristearin, followed by tripalmitin and trimyristin. The lowest amount of API, 1.2 wt.%, could be loaded into the Compritol particles. Because of the particle instabilities occurring during the drug loading procedure, the loading results may, however, have to be regarded with caution.

Due to the particle agglomeration or aggregation of the lipid suspensions during FENO loading, only the lipid nanoemulsions were further processed into dry powders in this study.

**Figure 6:**
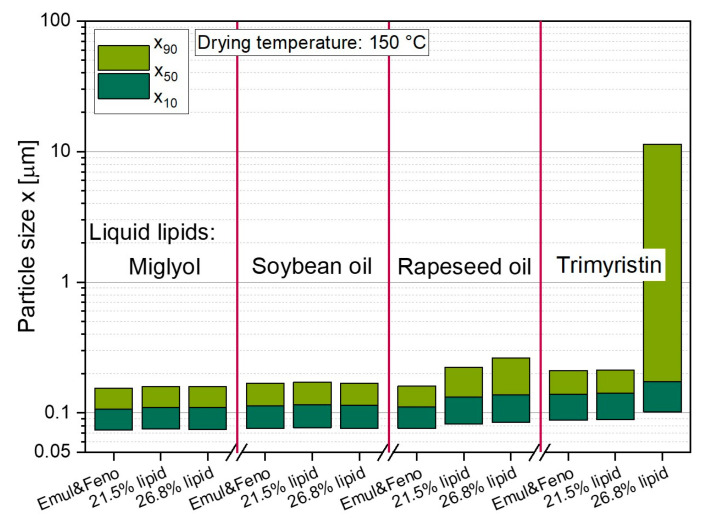
Particle sizes of lipid nanoemulsions loaded with FENO and of redispersed powders with lipid contents of 21.5 wt.% and 26.8 wt.%, respectively.

The results of the spray-dried lipid nanoemulsions indicate that after loading the lipid droplets with FENO, the nanoparticulate properties of the initial emulsions were maintained (Figure 6). Only a slight increase in particle sizes was observed, resulting in dispersions with a x_50_ between 107 nm and 138 nm. The two powders containing the API-loaded Miglyol and soybean oil emulsion did not show any increase in particle size after spray drying of the nanoemulsions, and the results also indicated that the lipid content in the powders did not affect the resulting particle sizes. For the powders in which rapeseed oil was embedded, a slight increase in droplet size of approx. 20 nm was observed. For rapeseed oil emulsions, it was also found that the x_90_ gradually increased with higher lipid contents in the dried powders. Coalescence of the droplets was evident when 26.8 wt.% of trimyristin droplets were embedded in the lactose/SDS matrix, while this phenomenon did not occur when the lipid content in the dried powders was lower.

Assuming an homogeneous distribution of the lipid droplets in the powders, it was calculated that up to 2.19 mg of FENO could be administered to the patient with a 100 mg powder formulation when the lipid carrier was prepared from Miglyol and the lipid content in the powder was 26.8 wt.%. If a tablet or capsule with a total weight of 800 mg made from this powder was considered, approx. 17.52 mg of FENO could be delivered. Slightly less FENO, 1.52 mg to 1.58 mg, could be administered with 100 mg if one of the other oils was chosen as the lipid carrier system.

### 3.5. Influence of Drying Temperature on Lipid Nanoparticles

The effect of the drying temperature on the redispersibility of the dried powders and on the efficiency of the process was first investigated in more detail in this section. Subsequently, the influence of the temperature on the state of the lipid particles was studied in more detail. All experiments were performed with PVA/SDS-stabilized lipid suspensions without API and with a lipid content of 21.5 wt.% in the lactose/SDS matrix.

#### 3.5.1. Particle Sizes and Process Evaluation

**Figure 7:**
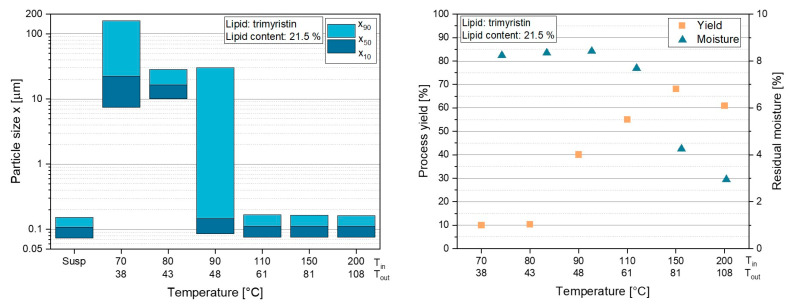
Influence of drying temperature T_in_ and the resulting T_out_ on the particle sizes after powder redispersion (**left**) and the process yield as well as residual moisture of the powders (**right**). The experiments were performed with a trimyristin suspension with 21.5 wt.% embedded lipid in the lactose/SDS matrix during drying.

To investigate the process performance as a function of the drying temperature, trimyristin nanosuspensions (x_50_ = 107 nm) were spray-dried at T_in_ between 70 °C and 200 °C. The resulting T_out_ ranged from 38 °C and 108 °C for these formulations (Figure 7).

No aggregation/agglomeration occurred during spray drying when the formulation was dried at temperatures of T_in_ = 110 °C or above (Figure 7, left). However, agglomeration/aggregation tendencies were observed when Tin was reduced to 90 °C, as indicated by the high x_90_-value. When the drying temperatures were further reduced to T_in_ = 80 °C and below, redispersion of the powders was no longer possible. Although dry powders were obtained at these drying temperatures, large agglomerates/aggregates were formed, which could no longer be properly redispersed in water.

To evaluate the cause for this behavior, the process yields as well as the residual moisture of the powders after spray drying were measured. With a process yield of 10 %, the lowest amount of dried powder was obtained at T_in_ of 70 °C and 80 °C (Figure 7, right). The yield gradually increased with higher T_in_ and, thus, the highest amount of dried powder was obtained at T_in_ = 150 °C with a process yield of approx. 69 %. However, the results also showed that the yield decreased again as soon as T_out_ was above the glass transition temperature of lactose (T_g_ = 101 °C) [28], as obtained for T_in_ = 200 °C with T_out_ = 108 °C. It became clear that in addition to the large agglomerates formed during spray drying of the trimyristin suspension at temperatures between T_in_ = 70 °C and 90 °C, the process yield in these experiments was well below 50 %. The residual moisture in the lipid-containing powders was determined in order to assess whether it could have an influence on the redispersibility as well as the process performance (Figure 7, right). The residual moisture was above 7 wt.% for all powders dried at temperatures of T_in_ = 110 °C and below. When the formulation was dried at higher temperatures, the water content was significantly reduced. The lowest moisture of 3 wt.% was obtained at the highest drying temperature at T_in_ = 200 °C (T_out_ = 108 °C). These results could not explain the agglomeration tendencies of the powders dried at low temperatures (T_in_ = 70 °C and 90 °C), as high water contents were also found in the powder dried at T_in_ = 110 °C, which did not show agglomeration during redispersion. To further investigate these phenomena, XRD measurements were performed with these powders.

**Figure 8:**
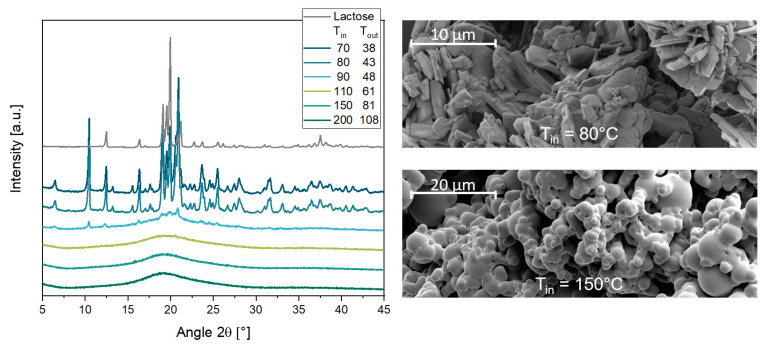
X-ray diffractograms of lactose starting material and trimyristin suspensions spray-dried at T_in_ between 70 °C and 200 °C (**left**); SEM images of lipid-containing powders dried at T_in_ = 80 °C (**right**, **top**) and T_in_ = 150 °C (**right**, **bottom**).

A significant influence of the drying temperature on the amorphous and crystalline state of the trimyristin-containing powders was observed (Figure 8, left). Compared to the lactose starting material, which exhibited characteristic reflections at, for example, approx. 12.5, 17, and 22° 2θ, the absence of these reflections indicated an amorphous state of the lactose matrix when the formulations were dried at temperatures of T_in_ = 110 °C and higher. However, a very pronounced crystalline structure was observed when the trimyristin suspension was dried at T_in_ = 70 and 80 °C. For the powder spray-dried at T_in_ = 90 °C, a combination of both physical states, crystalline and amorphous, was observed. SEM images of two powders dried at different temperatures (T_in_ = 80 °C and 150 °C) confirmed these findings (Figure 8, right). While a platelet-like structure of the lactose was identified in the crystalline sample dried at T_in_ = 80 °C, the lactose matrix particles dried at T_in_ = 150 °C had a spherical shape. These findings exhibited a direct correlation with the particle sizes after redispersion of the powders. Good redispersibility of the powders was obtained when the formulations were dried at T_in_ = 110 °C and higher, resulting in an embedding of the trimyristin particles in an amorphous lactose matrix. When the formulation contained crystalline lactose particles after drying, a poor redispersibility was obtained, as shown at T_in_ = 70 °C and 80 °C. Partially small particles but also large agglomerates were redispersed from the powders when trimyristin was embedded in a lactose matrix, which contained both crystalline as well as amorphous components (T_in_ = 90 °C). Correlations could be made with the previously used matrix material mannitol (Section 3.1.1), which also embedded the dispersions in a crystalline structure and did not allow a suitable redispersion of the lipid nanoparticles from the dried powders regardless of the addition of the surfactant SDS. In conclusion, drying temperatures between T_in_ = 110 °C and 200 °C were advantageous, as good redispersibility of the particles was achieved by embedding them in an amorphous lactose matrix and high process yields were obtained under these conditions.

#### 3.5.2. Melting of Lipid Particles during Spray Drying

In this section, the extent to which the particles in the spray-drying formulation melt during the drying process and the influence of T_in_ as well as T_out_ were investigated. The experiments were performed with a tristearin formulation stabilized with the additive HPMC and the surfactant SDS during high-pressure homogenization. A lipid content of 21.5 wt.% was embedded in the lactose/SDS matrix and T_in_ was set between 70 °C and 200 °C. The results presented above showed that the yield of the spray-drying process was temperature-dependent. During drying at lower temperatures, the deposition of the particles on the walls of the cyclone increased, which could theoretically lead to a higher temperature exposure of the particles than if they had been deposited directly in the glass vessel. Although the samples for the DSC measurements were taken from the glass vessel, it cannot be excluded that particles that initially adhered to the glass walls of the cyclone did not detach from the walls of the cyclone during the drying process. However, based on the experimental setup, it can be assumed that the maximum temperature to which the particles are deposited on either the walls of the cyclone or in the collection vessel is T_out_ (measured directly after the particles leave the drying camber).

**Figure 9:**
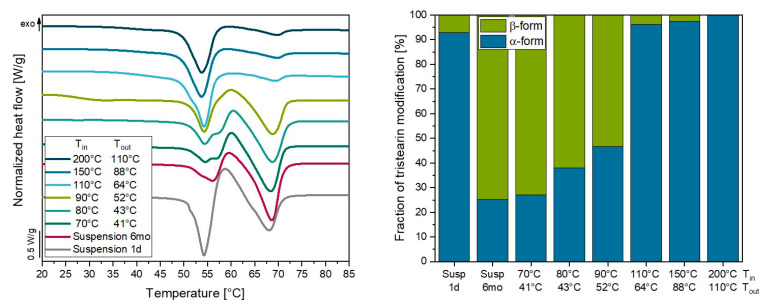
DSC heating curves of tristearin suspensions stabilized with HPMC/SDS during high-pressure homogenization measured one day (1 d) as well as 6 months (6 mo) after preparation and of tristearin-nanoparticle containing powders dried at different T_in_ (**left**); fraction of tristearin in the α- and β-form in the suspensions and the powders as calculated from the DSC curves (**right**).

As the temperatures inside the droplets cannot be measured during spray dying, the tristearin suspension was used as a tracer formulation for this purpose. After preparation of the tristearin suspension by high-pressure homogenization, the nanoparticles crystallized mainly in the metastable α-modification when the formulation was cooled in an ice bath for 30 min and afterward warmed to room temperature. An identification of the modification present in the formulations was possible based on the different melting temperatures of the α- and the β-modification, as shown in Figure 9 (left). The DSC heating curve of the tristearin suspension one day after its preparation (1 d) showed an endothermic peak at approx. 54 °C, indicating melting of the particles in the α-polymorph. An exothermic peak followed at approx. 58 °C, pointing to the recrystallization of previously molten particles into the stable β-polymorphic form. The following endothermic peak at approx. 68 °C represented the melting event of particles in the β-polymorph. From this DSC measurement, the fraction of particles in the α- and the β-modification was calculated according to Joseph et al. [33] (Figure 9, left). One day after preparation of the suspension (t = 1 d), approx. 93% of the particles were in the α-polymorph and approx. 7 % in the more stable β-form. Particle transformation was detected when the tristearin suspension was stored over 6 months (at 20 °C), after which the fraction of tristearin particles in the β-modification had increased clearly to approx. 75 %. To evaluate the influence of the spray-drying temperature on the particles, the stored tristearin suspension (t = 6 mo) was spray-dried and the obtained powders were directly measured by DSC to determine the modification of the particles therein.

Drying the suspension at the lowest temperature, T_in_ = 70 °C, resulting in T_out_ = 41 °C, did not significantly change the fraction of tristearin particles in the α- or β-modification. A slight decrease in the fraction in the β-modification was observed when the tristearin formulation was dried at T_in_ = 80 °C and 90 °C (T_out_ = 43 °C and 52 °C, respectively). It was assumed in this study that a decreased fraction of particles in the stable β-polymorphic form could only occur if the particles melted during the drying process and re-crystallized in the metastable α-modification. Apparently, only a small to moderate fraction of particles melted during the drying process and re-crystallized into the metastable α-form at process temperatures up to 90 °C. A significant increase in the fraction of tristearin particles in the α-modification was observed when the suspension was spray-dried at T_in_ = 110 °C and higher, resulting in T_out_ of at least 64 °C. It was concluded that at these process temperatures (almost), all particles must have melted during the spray-drying process and afterward crystallized in the α-polymorphic form. Previous studies already attempted to estimate the temperature exposure of the particles during the drying process, as direct measurements have not been possible so far. As for the results obtained in this study, the temperature exposure was derived from the fraction of α- and β-modification in the tristearin-containing powders. It seemed to be higher than previously expected by other research groups [15,16,17], as the DSC curves indicated that the majority of the nanoparticles in the β-modification melt at approx. 68 °C (heating rate 10 K min^−1^, similar results were obtained also at lower heating rates (data not shown)), determined at the maximum of the melting curve of the suspension (Figure 9, left). Computer simulations examining the path of the sprayed particles through the spray-drying tower during drying showed that the particles did not only move on the centerline but could also recirculate in the tower, which would explain the temperature exposure of particles above T_out_ [34,35]. Generally, however, in this study, it was assumed that the described phenomenon may depend on the type of spray dryer used as well as on process parameters. In the current study, no recirculation of the particles was visually detected, but it could not be ruled out either. In this regard, further experiments should be conducted to determine the actual temperatures the particles are exposed to during the drying process. Lipid nanoparticles that exhibit peculiar melting and recrystallization phenomena (e.g., also in dependence on particle size) may serve as valuable probes for this purpose.

## 4. Conclusions

Spray drying is a very suitable method to embed lipid nanodispersions—lipid nanosuspension as well as -emulsions—in a solid matrix. In this study, the nanoparticulate properties of the dispersions could be maintained when the lipids were embedded in a matrix containing lactose in combination with the surfactant SDS. For tristearin particles, it was also shown that excipients used for the particle stabilization during high-pressure homogenization had an effect on the quality of the final product.

Redispersible API-containing powders could be obtained when oils were used as carrier systems in the nanodispersions. With an FENO load of up to 8.2 wt.% in the Miglyol matrix, up to 2.19 mg of FENO could be processed per 100 mg of dried powder, while maintaining the nanoparticulate properties of the nanoemulsion. Similarly good redispersibility was achieved for the FENO-loaded nanoemulsions with the other oils under investigation such as soybean and rapeseed oil.

Low spray-drying temperatures led to the formation of crystalline lactose during the drying process, resulting in poor redispersibility of the nanoparticulate lipid from the dried powders. When inlet temperatures of 110 °C or higher were selected, the lactose was in an amorphous state after drying and the nanoparticulate properties could be maintained. Particle melting was observed during spray drying by applying a tristearin formulation. At T_in_ = 110 °C, which resulted in T_out_ = 64 °C, almost all particles melted during the drying process. The temperature the lipid nanoparticles were exposed to seemed to be higher than the outlet temperature, because the melting temperature of the tristearin nanoparticles in the suspension (β-modification) was determined to be approx. 68 °C. Until now, it was only possible to estimate the temperature to which the particles were exposed to during the spray-drying process. With the lipid formulation developed in this study, the temperature exposure could directly be measured via melting of the lipid particles during the process.

## Figures and Tables

**Table 1:** Viscosity of lipid nanosuspensions after high-pressure homogenization and of the formulations prepared directly before spray drying, containing the suspension and the matrix former solution, for the two particle stabilization systems (during high-pressure homogenization): PVA/SDS and HPMC/SDS.

	Particle Formulation	
**Formulation**	PVA/SDS	HPMC/SDS
**Lipid Nanosuspension**	14.6 mPas	103.0 mPas
**Drying Formulation with Lipid Content**:		
21.5 wt.%	1.5 mPas	2.5 mPas
31.5 wt.%	1.8 mPas	5.4 mPas

## Data Availability

Not applicable.
